# Analysis of Microbial Community Heterogeneity and Carbon Fixation Capabilities in Oil-Contaminated Soils in Chinese Onshore Oilfields

**DOI:** 10.3390/microorganisms12112379

**Published:** 2024-11-20

**Authors:** Jiayu Song, Yakui Chen, Yilei Han, Yunzhao Li, Zheng Liu, Xingchun Li, Diannan Lu, Chunmao Chen

**Affiliations:** 1 State Key Laboratory of Petroleum Pollution Control, Beijing 102206, China; songjiayu@cnpc.com.cn (J.S.); li-xingchun@cnpc.com.cn (X.L.); 2 CNPC Research Institute of Safety and Environmental Technology, Beijing 102206, China; 3 Department of Chemical Engineering, Tsinghua University, Beijing 100084, China; chenyk1006@126.com (Y.C.); liuzheng@tsinghua.edu.cn (Z.L.); 4 College of Chemical Engineering and Environment, China University of Petroleum-Beijing, Beijing 102206, China; lyz18780270046@163.com (Y.L.); c.chen@cup.edu.cn (C.C.)

**Keywords:** oil-contaminated soils, microbial diversity, carbon fixation, correlation network, petroleum hydrocarbon degradation, metabolic pathways

## Abstract

This study selected 27 soil samples from four representative horizontally distributed onshore oilfields in China to explore the diversity of soil microbial communities and their carbon fixation capacity, with a focus on the potential interaction between pollution and carbon fixation under oil pollution stress. The analysis of the soil physicochemical properties and microbial community structures from these oilfield samples confirmed a clear biogeographic isolation effect, indicating spatial heterogeneity in the microbial communities. Additionally, the key factors influencing microbial community composition differed across regions. The dominant bacterial phyla of soil microorganisms under soil pollution stress were Proteobacteria, Actinobacteriota, Chloroflexi, Acidobacteriota, Firmicutes, Bacteroidota, and Gemmatimonadota. A correlation network analysis identified *Immundisolibacter*, *Acinetobacter*, *Blastococcus*, *Truepera*, and *Kocuria* as key players in the microbial network, with most showing positive correlations. The results of the KEGG database functional annotation showed that degradation and carbon fixation metabolic pathways coexist in soil samples and maintain a balanced relative abundance. These metabolic pathways highlight the functional diversity of microorganisms. Among them, prokaryotic and eukaryotic carbon fixation pathways, along with benzoate degradation pathways, are predominant. These findings establish a theoretical basis for further exploration of the synergistic mechanisms underlying pollution reduction and carbon sequestration by microorganisms in petroleum-contaminated soils.

## 1. Introduction

Oil pollution is prevalent worldwide, particularly in areas associated with oil extraction, transportation, and processing [1,2,3]. This pollution alters soil structure, reduces soil fertility, disrupts ecosystems, and poses significant risks to human health, thereby seriously threatening environmental health and ecological balance. It has emerged as an urgent environmental issue that requires immediate attention [4].

Long-term exploitation and leakage in China’s onshore oilfields have caused severe soil pollution, posing significant threats to the ecology and agricultural production [5,6]. Although traditional physical and chemical remediation methods can be effective, they are costly and may cause secondary pollution [7,8]. In recent years, bioremediation technologies, especially using microorganisms, have garnered considerable attention due to their environmental friendliness, cost-effectiveness, and high efficiency [9,10]. This technology employs microorganisms to degrade petroleum pollutants, thereby aiding in the restoration of soil health. However, the diversity and functional capabilities of microorganisms in contaminated soils can vary significantly, necessitating further systematic research to optimize these processes [11,12,13].

The soil carbon pool is the largest carbon reservoir in terrestrial ecosystems. Enhancing soil carbon storage is both an economically feasible and environmentally friendly approach to carbon sequestration, making it a crucial strategy for mitigating global climate change. Microorganisms, as key drivers of the soil carbon cycle, play a vital role in processes such as carbon degradation, methane metabolism, and carbon fixation. A deeper understanding of the functions and roles of microbial communities in the carbon cycle is essential for comprehending their response, adaptation, and feedback mechanisms to global climate change. Regarding petroleum-contaminated soils, numerous studies have focused on exploring the diversity of microorganisms in contaminated soils [14,15], their pollutant degradation capabilities and mechanisms [16,17,18], their effects on plant growth [19], and their capacity for carbon fixation [20,21]. Studies have demonstrated that oil pollution significantly influences the composition and metabolic functions of microbial communities. For instance, certain bacteria and fungi exhibit strong oil degradation abilities, becoming dominant in contaminated soils [22,23]. *Cyanobacteria* [24], *Nitrosomonas* [25], *Chromatium* [26], *Rhizobium* [27], and *Blastococcus* [28] have been shown to have carbon fixation capabilities. However, research on the interaction between microbial pollutant reduction and carbon fixation is still limited. In summary, although existing studies have elucidated some basic aspects of microbial diversity and carbon fixation in contaminated soils, comprehensive research on the microbial community structure and carbon fixation capacity of contaminated soils in onshore oilfields in China is still insufficient.

This study utilized metagenomic and high-throughput sequencing technologies to systematically assess the diversity of microorganisms in contaminated soils from typical onshore oilfields in China. It analyzed the distribution patterns of microbial communities across different geographical locations and environmental conditions and investigated their carbon fixation capabilities in polluted environments, and further revealed the interactive relationship between pollution reduction and carbon fixation. These research results lay the groundwork for further exploration of the synergistic mechanisms of pollution reduction and carbon fixation under soil pollution stress, provide a theoretical foundation for developing innovative microbial remediation technologies, and offer a scientific basis for formulating effective soil ecological restoration strategies, thereby enhancing soil health and carbon fixation efficiency.

## 2. Materials and Methods

### 2.1. Study Sites and Soil Sampling

Soil samples were collected from typical onshore oilfields in China in June 2023, designated as C, NW, SW, and E. All four oilfields are in the middle to late stages of development and exhibit significant variations in altitude and climate conditions (Table 1). The total number of representative soil samples and descriptions by location are as follows: Eastern Oilfield (E, 1–11), Central Oilfield (C, 1–7), Northwestern Oilfield (NW, 1–4), and Southwestern Oilfield (SW, 1–5). Samples were collected from the surface soil (0–20 cm) of the four oilfields. We established a 20 m × 20 m sampling area and used the five-point sampling method to collect equal amounts of soil from each designated point. We thoroughly mixed the soil from all points to obtain a representative sample for each location. Using a sterile iron shovel, we collected approximately 500 g of the mixed soil and placed it into a sterile sealed bag, and any animal and plant remains, as well as gravel, were removed. The samples were then stored in a 4 °C icebox and transported to the laboratory, where they were divided into two parts. One part was naturally air-dried and passed through a 2 mm sieve for the determination of soil physicochemical indicators and total petroleum hydrocarbons, while the other part was stored at −80 °C for a soil microbial diversity analysis and metagenomic sequencing.

### 2.2. Determination of Soil Physical and Chemical Properties

The soil physicochemical properties were determined following the methods established in previous studies [29], including soil particle composition, pH, moisture content, electrical conductivity, soil organic carbon (SOC), ammonium nitrogen, nitrate nitrogen, total nitrogen (TN), and total phosphorus (TP). The results of the physicochemical property tests for the soil samples are presented in Table S1.

### 2.3. Determination of Total Petroleum Hydrocarbon

Total petroleum hydrocarbons (TPHs, mg/kg) were quantified using gas chromatography (7890A, Agilent Technologies, Nantong, Jiang Su, China). A 10.0 g sample was weighed in a mortar, mixed with an appropriate amount of diatomaceous earth, and ground to a homogeneous quicksand state. The sample was then extracted using an accelerated solvent extractor (ASE-350, Thermo Fisher Scientific, Waltham, MA, USA) under the following conditions: pressure of 1200 psi, temperature of 100 °C, static extraction for 5 min, elution volume of 60%, nitrogen purging for 60 s, 2 extraction cycles, and with an acetone-*n*-hexane mixed solvent (1:1 *v*/*v*) as the extractant. The extract was washed twice with 100 mL of ultrapure water, and the upper organic phase was retained. After dehydration with anhydrous sodium sulfate, the extract was concentrated to 1.0 mL by rotary evaporation (RV10, IKA, Breisgau, Baden-Württemberg, Germany) to prepare the sample for analysis. TPH content was determined using a gas chromatograph (7890A, Agilent Technologies, Santa Clara, CA, USA) equipped with a *ϕ* 0.32 mm × 30 m × 0.25 μm capillary column (DB-5MS, Agilent Technologies) and a flame ionization detector (FID). The instrument conditions were as follows: injection port temperature at 300 °C, detector temperature at 325 °C, column temperature at 320 °C. The oven temperature program was as follows: initial temperature of 50 °C held for 2 min, increased to 230 °C at a rate of 40 °C/min, and then to 320 °C at a rate of 20 °C/min and held for 20 min. The carrier gas flow rates were 99.999% high-purity nitrogen at 1.5 mL/min, 99.99% high-purity hydrogen at 30 mL/min, and air at 300 mL/min. Non-split injection mode was used with an injection volume of 1 μL. The total petroleum hydrocarbon content results are presented in Table S2.

### 2.4. Microbial Analysis

Total DNA was extracted from 0.25 g of soil samples using a DNeasy PowerSoil^®^ Pro Kit (Qiagen, Hilden, Germany) [30], following the manufacturer’s instructions. The concentration and purity of the extracted DNA were measured with a NanoDrop 2000 micro-volume spectrophotometer (Thermo Fisher Scientific, Waltham, MA, USA) [31], and the integrity of the DNA was assessed by 1% agarose gel electrophoresis.

For microbial diversity analysis, 16S rRNA gene sequencing was employed. The highly variable regions of the 16S rRNA gene, such as the V3–V4 region, were amplified by PCR [32] using the primers 338F (5′-ACTCCTACGGGAGGCAGCAG-3′) and 806R (5′-GGACTACHVGGGTWTCTAAT-3′) [33]. The PCR amplification was carried out in a 20 μL reaction mixture, consisting of 4 μL of 5X FastPfu Buffer, 2 μL of 2.5 mmol/L dNTPs, 0.8 μL of each primer, 0.4 μL of FastPfu Polymerase, 0.2 μL of BSA solution, 1 μL of template DNA (approximately 10 ng), and sterile ddH_2_O up to 20 μL. The PCR conditions were as follows: initial denaturation at 95 °C for 3 min, followed by 30 cycles of denaturation at 95 °C for 30 s, annealing at 53 °C for 30 s, and extension at 72 °C for 30 s, with a final extension at 72 °C for 5 min. The PCR products were purified using an AxyPrep DNA Gel Extraction Kit (Axygen Scientific, Union City, CA, USA) [34] and their quality was verified using an Agilent 2100 Bioanalyzer (Agilent Technologies, Santa Clara, CA, USA). The sequencing of microbial diversity was performed by Biomarker Technologies Corporation, Beijing, China, and the extracted DNA was sequenced using the Illumina MiSeq PE300 platform (Illumina, San Diego, CA, USA).

Metagenomic sequencing was conducted using a Covaris ME220 Focused-Ultrasonicator (Covaris Inc., Woburn, MA, USA) to fragment DNA, targeting a fragment length of 400 bp [35]. Library preparation and bridge PCR amplification were carried out with a NEXTFLEX™ Rapid DNA-Seq Kit (PerkinElmer, Austin, TX, USA) to obtain the DNA fragment sequence. Sequencing was performed by Meiji Biopharmaceutical Technology Co., Ltd (Shanghai, China). on the Illumina NovaSeq 6000 platform (Illumina, San Diego, CA, USA), employing paired-end sequencing to ensure high coverage and high-resolution data [36].

### 2.5. Data Analysis

Sequencing data were processed using the QIIME2 platform (version 2024), with quality control and denoising conducted via the DADA2 plugin (https://qiime2.org). Circular diagrams were generated using Circos (version 0.67-7), while Venn diagrams were created by R (version 3.3.1). Stacked bar charts were produced using R (version 3.3.1). Species correlation network diagrams were constructed with Cytoscape 3.7.1. Metabolic pathway diagrams were developed using Origin 2022.

## 3. Results and Discussion

### 3.1. Soil Physical and Chemical Properties and Pollutant Indicators

**Figure 1:**
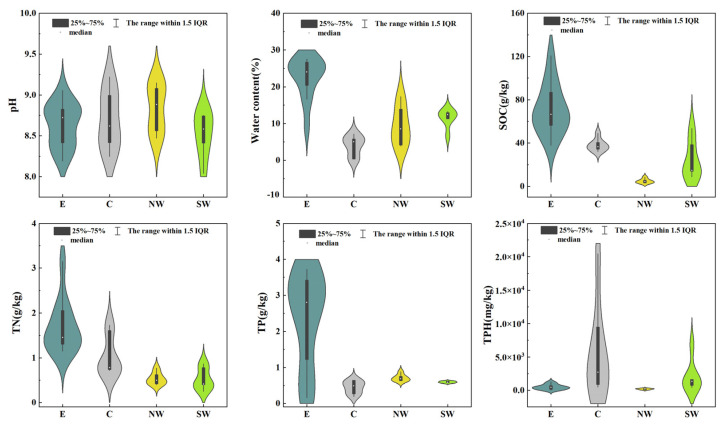
Physicochemical indexes and petroleum hydrocarbon content of contaminated soil in each oilfield.

Figure 1 displays the physical and chemical properties and pollutant values and distribution of soil samples in the four regions, including pH, water content, soil organic carbon (SOC), total nitrogen (TN), total phosphorus (TP), and total petroleum hydrocarbons (TPHs). It is shown in Figure 1 that the soil samples from the four regions were all alkaline. Additionally, the water content, SOC, TN, and TP levels in the samples from region E were significantly higher than those in the other regions, which may be attributed to the enhanced soil structure and water retention capabilities in organic-rich soils, which support more robust microbial activity [37]. Kögel-Knabner showed that the organic components of plants or microorganisms can be used as soil organic objects to enhance soil microorganisms [38]. Such conditions provide a favorable environment for microorganisms, thereby promoting soil health and ecosystem stability [39,40]. Conversely, the lower water content and SOC in the northwest samples likely limit microbial survival and activity, leading to reduced microbial diversity [41]. The elevated total petroleum hydrocarbons (TPHs) in the central region suggest contamination by petroleum. This not only alters the soil’s physicochemical properties but also potentially exerts a negative impact on the microbial community [42,43]. These observations suggest that water content, SOC and TPH indicators may have a significant impact on the structure and function of soil microbial communities [44,45].

### 3.2. Analysis of Microbial Community Diversity and Family-Level Composition

**Figure 2:**
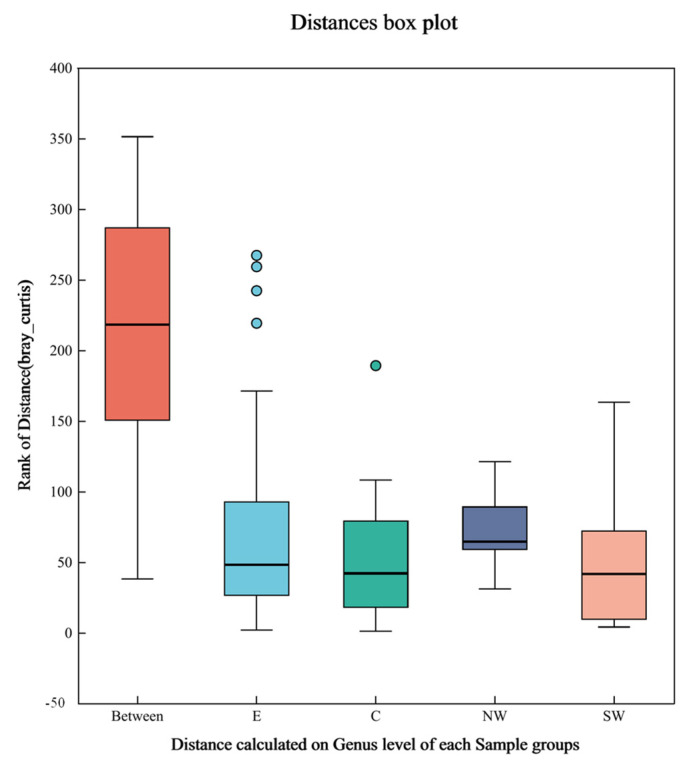
Boxplot of Bray–Curtis distance.

Figure 2 illustrates the differences in microbial community structures both between and within different soil sample groups. In this figure, “Between” denotes the distance between groups, while E, C, NW, and SW represent the distances within groups. The Bray–Curtis distance between groups (Between) was significantly higher than within the groups (E, C, NW, and SW), indicating notable differences in the soil microbial communities across different geographical regions at the genus level. This suggests that geographical location is a critical factor influencing the structure of soil microbial communities.

The relatively low distance values within the groups, averaging about 50 in the box plots, indicated that the microbial communities within each region are quite consistent. This consistency reflects the adaptability of soil microbial communities to local environmental conditions. These interregional differences are likely associated with regional variations in soil physicochemical properties, such as pH, water content, soil organic carbon content, total nitrogen, and total phosphorus levels [44]. For instance, higher pH and organic matter contents generally support greater microbial diversity, whereas high levels of pollutants, such as total petroleum hydrocarbons and heavy metals, can limit microbial diversity and function [46].

Additionally, the high intergroup distances suggested a significant biogeographic isolation effect among the soil microbial communities across different regions. This effect is likely due to varying soil conditions and environmental pressures that drive differentiation in the evolution and ecological functions of microbial communities [47,48].

**Figure 3:**
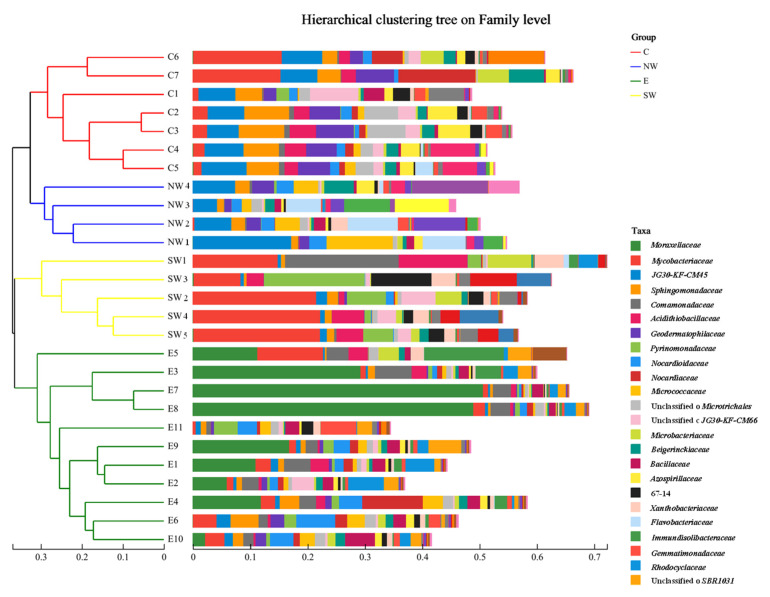
Hierarchical clustering tree on family level.

Figure 3 presents the microbial community structure of the soil samples from different regions at the family level. The hierarchical clustering tree categorizes the samples into several groups, each with a similar community composition. For example, samples within the SW group cluster closely together. The E, C, NW, and SW groups exhibit distinct community structure characteristics. The accompanying bar chart illustrates the relative abundance of each family in the samples from different regions. The dominant families in E and SW groups were *Moraxellaceae* and *Mycobacteriaceae*, respectively, while in C and NW groups, *Sphingomonadaceae*, *Geodermatophilaceae*, and *Microbacteriaceae* were more abundant. These findings align with previous research, which highlights the importance of spatial heterogeneity in the soil environment in shaping the structure of microbial communities [49,50].

### 3.3. Analysis of Microbial Community Structure and Diversity

The Alpha diversity index is a comprehensive measure used to evaluate the diversity and richness of soil microbial communities [51]. The Simpson index assesses community evenness, with larger values indicating a more balanced distribution of microbial species [52]. The Ace index estimates species richness, where higher values reflect a greater number of species in the sample [53]. Changes in the Alpha diversity of contaminated soil samples from different oilfield areas are detailed in Table S3. As shown in Table S3, the coverage index for all samples was close to one, indicating high data reliability. Significant differences in the Alpha diversity index were observed among contaminated soil samples from different oilfield areas. Notably, samples E7 and E8 exhibited higher Simpson and Ace indices, suggesting that these areas have more balanced and species-rich microbial communities. This could be attributed to favorable soil physicochemical properties and specific pollution conditions [39]. In contrast, samples C7 and NW1 showed lower diversity and evenness, likely due to higher pollution pressures or suboptimal soil conditions [54,55].

**Figure 4:**
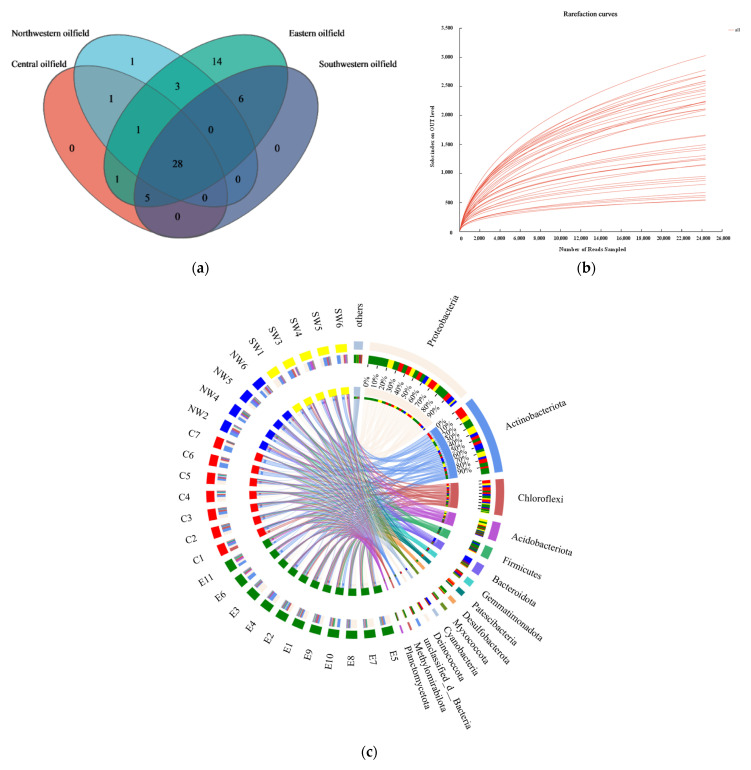
Analysis of soil microbial community sharing, diversity, and network structure. (**a**) Venn diagram of microbial taxa in different oilfield areas; (**b**) dilution curve of all sample sequencing results; (**c**) circular network diagram of microbial communities in petroleum-contaminated soils from different oilfields (phylum level).

Figure 4 provides a detailed depiction of the sharing, diversity, and functional network of soil microbial communities. Figure 4a illustrates the shared and unique taxa of the microbial communities across different regions. There are 28 shared taxa in the four regions, which not only reflect wide adaptability and distribution but also may be involved in soil pollution reduction and carbon sequestration processes. The Eastern (E) oilfield hosts the most unique taxa, which may be related to the rich nutrient status or unique pollutant degradation ability of the region. Figure 4b displays the rarefaction curves for each soil sample, showing the accumulation of operational taxonomic unit (OTU) richness with increased sequencing depth. As the sequencing depth increases, the number of OTUs in each sample stabilizes, indicating that further sequencing may yield diminishing returns in discovering new OTUs [56,57]. These findings suggest that the current sequencing strategy is effective, though higher sequencing depth or enrichment strategies might still be necessary for detecting rare taxa or specific functional groups. Figure 4c depicts the complex structure and functional relationships within the microbial community. High-abundance taxa such as Proteobacteria and Actinobacteriota exhibit broad environmental adaptability. The distribution and interconnections of these taxa across different samples highlight the multifunctionality of the microbial community within the ecosystem [58] and the potential involvement of key pollution reduction and carbon fixation processes. For example, some members of Proteobacteria are known to be involved in the degradation of organic pollutants [59], while some members of Actinobacteriota may play a role in carbon fixation [60], such as promoting the formation of soil organic matter through their metabolic activities. The connections in the circular network diagram highlight the interactions between microbial communities, which are not only critical for soil health but also play an important role in ecosystem services, especially pollution reduction and carbon sequestration [61]. For example, symbiotic relationships between microorganisms may enhance their ability to degrade pollutants [62], while the complementary functions of certain microbial communities jointly promote soil carbon fixation [63].

### 3.4. Correlation Analysis Between Environmental Factors and Soil Community Structure

**Figure 5:**
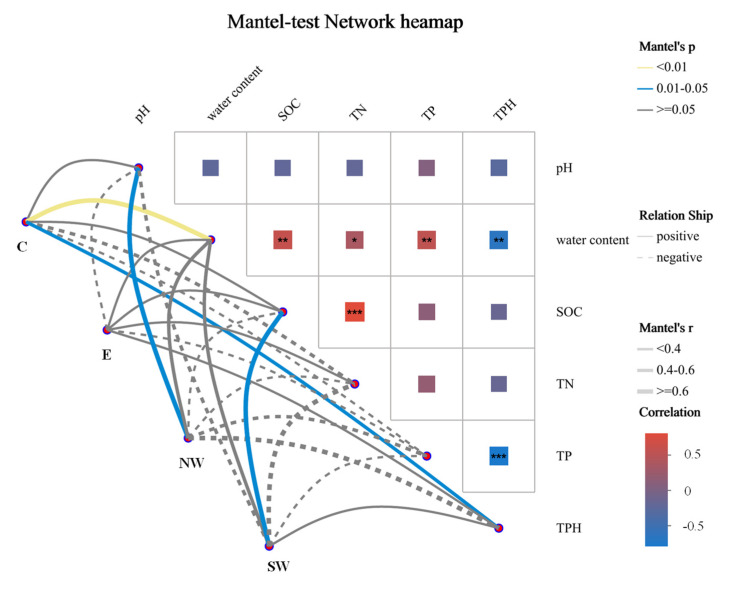
Environmental drivers of soil microbial community composition. Note: The lines in the figure represent the correlation between the community and environmental factors. The thickness of the lines indicates the magnitude of the correlation. The Mantel r method was used for drawing. The heat map represents the correlation between environmental factors. Different colors represent positive and negative correlations. The depth of the color represents the magnitude of the positive and negative correlations. The asterisks in the color blocks represent significance. * 0.01 < *p* ≤ 0.05, ** 0.001 < *p* ≤ 0.01, *** *p* ≤ 0.001.

The microbial community structure in the four regions was found to be correlated with various environmental factors. Figure 5, the Mantel test identified water content and TPHs as the most significant factors influencing the composition of soil microbial communities in region C, indicating a strong correlation between these factors and the microbial communities in that region. In the NW and SW regions, pH and SOC were the most significant factors, respectively, suggesting that adjusting the pH could effectively alter the microbial community composition in the NW region. These results demonstrated that different regions are influenced by distinct environmental factors that affect the composition and dynamics of soil microorganisms. Water content, SOC, pH, and pollutant indicators are likely key drivers of microbial changes in petroleum-contaminated soils.

### 3.5. Correlation Network Analysis of Potential Pollution-Reducing and Carbon-Fixing Microorganisms

**Figure 6:**
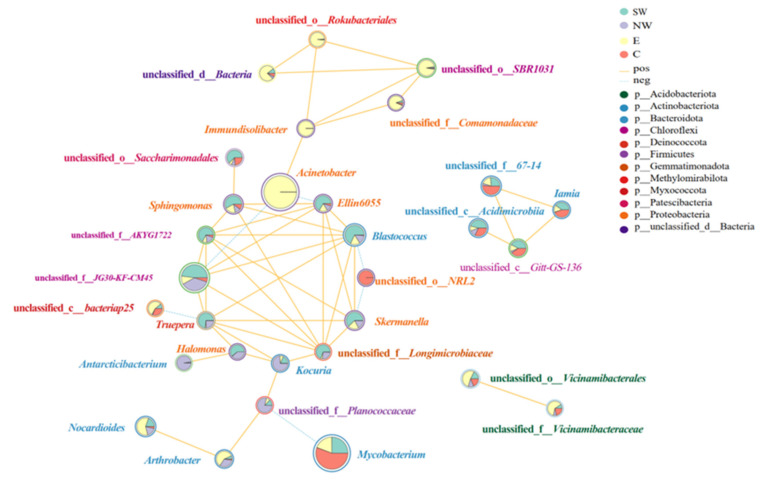
Correlation network of microorganisms in China’s onshore oilfield soils (genus level). Note: Each node represents a genus. The size of the node indicates the abundance of the species. Different colors represent different species. The color of the connecting line indicates a positive or negative correlation. Yellow indicates a positive correlation, and blue indicates a negative correlation. The thickness of the line indicates the size of the correlation coefficient. The thicker the line, the higher the correlation between species. The more lines there are, the closer the connection between the species and other species.

To investigate the potential interactions among soil microbial species in China’s onshore oilfields, the top 50 species based on total abundance were selected. Species with a relative coefficient greater than 0.75 and a significance level of *p* < 0.05 were retained for further analysis. This screening yielded 30 genus-level species across 13 phyla. The network node properties of these 30 genus-level species are detailed in Table 2, and the correlation network is illustrated in Figure 6.

The network node attributes include connectivity, degree centrality, the closeness coefficient, and betweenness centrality, which reflect the role and importance of each genus in the microbial network. Connectivity refers to the number of connections a node has with other nodes; a higher connectivity indicates greater importance within the network. Degree centrality directly measures a node’s centrality; a higher value signifies a more crucial position. The closeness coefficient reflects the average shortest path length between a node and other nodes; higher values denote proximity to the network center. Betweenness centrality represents the proportion of the shortest paths passing through a node; higher values indicate the node’s role as a bridge in the network.

As detailed in Table 2, genera such as *Immundisolibacter*, *Acinetobacter*, *Blastococcus*, *Truepera*, unclassified_f*_Longimicrobiaceae*, *Kocuria*, and *Ellin6055* exhibited high connectivity, degree centrality, closeness coefficients, and betweenness centrality. This suggests that these bacterial genera play pivotal roles in the microbial network. *Immundisolibacter* [64,65,66] and *Acinetobacter* [67,68,69] are extensively involved in the degradation of pollutants like petroleum hydrocarbons and polycyclic aromatic hydrocarbons, playing significant roles in environmental pollution mitigation. unclassified_f*_JG30-KF-CM45*, *Blastococcus* [70,71], and *Truepera* participate in carbon fixation and resource allocation, enhancing carbon cycling within the ecosystem. *Sphingomonas* not only has the ability to degrade PAHs [72], but also has a certain carbon fixation potential, making it a dominant genus in carbon fixation. *Antarcticibacterium*, with high betweenness centrality, is crucial for information or resource transfer between different microbial communities and may play a key role in pollution reduction and carbon fixation processes in the environment.

Figure 6 shows the relationship network diagram of key bacterial genera. The network diagram contains 30 nodes and 50 links, of which 44 are positively correlated (orange lines) and 6 are negatively correlated (blue dashed lines). Positively correlated connections indicate synergies between bacterial genera, such as complementarity in resource utilization and metabolic pathways. These interactions promote the functional diversity and stability of microbial communities. For instance, *Immundisolibacter* and *Acinetobacter* have been shown to be widely involved in the degradation of pollutants such as petroleum hydrocarbons and polycyclic aromatic hydrocarbons and play an important role in reducing environmental pollution [64,65,66,67,68,69]. The synergy between *Immundisolibacter* and *Acinetobacter* facilitates more effective decomposition of complex organic pollutants, thereby enhancing the resilience of soil microbial communities in polluted environments. Similarly, the interaction between *Nocardioides* and *Arthrobacter* not only excels in pollution reduction but also makes significant contributions to carbon fixation, which is essential for soil health and ecosystem services. By examining the complex network relationships within the microbial community, these microorganisms can be leveraged more effectively for environmental remediation and ecological protection. Understanding these intricate interactions provides a scientific basis for optimizing the application of microbial communities in pollution degradation and carbon sequestration.

### 3.6. Potential Microbial Metabolic Pathways for Pollution Reduction and Carbon Fixation in Onshore Oilfield Soils in China

The previous conclusions were further substantiated by analyzing the relative abundance of pollutant degradation and carbon fixation pathways. In this study, metagenomic sequencing was conducted on 27 representative soil samples, and the KEGG database was used to annotate the functional gene composition and metabolic pathways in these samples. Six primary metabolic pathways were identified: cellular processes, environmental information processing, genetic information processing, human diseases, metabolism, and organismal systems.

Table S4 presents the relative abundance of these primary metabolic pathways in the soil samples. It is evident from the table that the metabolism pathway has the highest relative abundance, varying between 76.5 and 80.5%, indicating that metabolic functions predominate in the microbial community. The annotated secondary metabolic pathways within the metabolism category focus on pathways related to pollution reduction and carbon fixation, including aromatic hydrocarbon degradation, complex organic compound degradation, and carbon fixation pathways.

**Figure 7:**
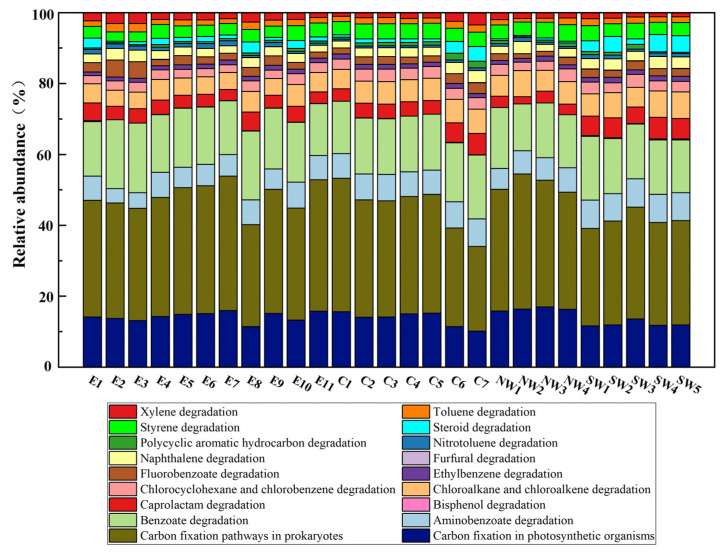
Relative abundance of pollutant degradation and carbon fixation pathways in soil samples.

Figure 7 shows the relative abundance of various metabolic pathways in different samples. Each bar represents a sample, with different colors corresponding to different pathways. The data indicated that these microbial communities possess diverse functional capabilities, with significant variation in the abundance of different pathways. For instance, the figure highlights that benzoate and polycyclic aromatic hydrocarbon (PAH) degradation accounts for a substantial proportion in many samples, underscoring the crucial role of microbial communities in pollution reduction and metabolic processes. Notably, carbon fixation pathways in prokaryotes and photosynthetic organisms (represented by light blue and dark blue, respectively) are prominently featured at many sampling points. This indicates that these microbial communities are actively engaging in carbon fixation while simultaneously degrading pollutants.

The coexistence of multiple functional pathways emphasizes the dual role of these microbial communities in pollution reduction and carbon cycling. Overall, these data underscore the functional diversity and ecological importance of microbial communities in environmental remediation and ecosystem stability. They play a vital role in both pollutant degradation and carbon fixation, demonstrating their versatility and supporting their potential use in bioremediation strategies and carbon management practices.

In recent years, the use of highly efficient degradation microorganisms and carbon fixation microorganisms for pollutant remediation and enhancement in soil carbon storage capacity has garnered significant attention from researchers. Tao et al. (2023) confirmed that microorganisms are the key to soil carbon storage [73]. *Pseudomonas*, *Bacillus megaterium*, and *thermophilic bacteria* have been confirmed to have the dual functions of pollution reduction and carbon fixation [74,75,76]. Li et al. isolated and characterized carbon-fixing bacteria, such as *Rubrivivax gelatinosus* and *Methylosinus trichosporium*, from petroleum-contaminated soils [77]. Studies have shown that carbon fixation efficiency can be significantly improved through genetic engineering [78], the optimization of culture conditions [79], the establishment of symbiotic systems [80,81], and synthetic biology [82]. Mao et al. (2021) explored carbon fixation efficiency from the perspective of functional genes [83], and few researchers paid attention to the relationship between pollution reduction and carbon fixation. Starting from pollution samples of onshore oilfields in China, we confirmed that the microbial community exhibits spatial specificity and functional diversity. Our findings reveal that soil microorganisms possess both pollution reduction and carbon fixation capabilities, maintaining a balanced relative abundance. This discovery fills the gap in research on the correlation between microbial pollution reduction and carbon fixation under oil pollution stress and lays a solid foundation for subsequent studies on the underlying metabolic mechanisms.

## 4. Conclusions

In this study, we systematically analyzed the microbial diversity and carbon fixation capacity of onshore oilfields in China. The results indicated a significant spatial heterogeneity in the structure of microbial communities, which were primarily dominated by Proteobacteria and Actinobacteria. The correlation between environmental factors and soil community structure reveals that the key factors influencing soil microbial community composition vary across different regions. A microbial network analysis revealed close interactions among different microbial genera. Notably, the synergistic effect between *Immundisolibacter* and *Acinetobacter* facilitated a more efficient decomposition of complex organic pollutants, while the interaction between *Nocardioides* and *Arthrobacter* contributed significantly to both pollution reduction and carbon fixation. This supports the theory that microbial communities achieve pollutant degradation and carbon fixation through metabolic synergy. Functional annotation using the KEGG database showed that both petroleum hydrocarbon degradation and carbon fixation metabolic pathways coexist in the soils of onshore oilfields in China. The relative abundance of carbon fixation pathways was balanced with that of degradation pathways. The dominant pathways included prokaryotic carbon fixation, eukaryotic carbon fixation, and benzoate degradation. These findings provide a theoretical basis for future studies on the molecular mechanisms underpinning the synergistic effects of microbial pollution reduction and carbon fixation. Additionally, they offer practical guidance for the development and optimization of microbial synergistic remediation technologies.

## Figures and Tables

**Table 1:** Climate information of the four studied oilfields in China.

Site	Latitude and Longitude	Climate Type	Average Altitude/m	Annual Mean Temperature/°C	Annual Average Precipitation/mm
E	38°43′00″ N 117°30′00″ E	Monsoon semi-humid	<5	15	550–600
C	37°9′53″ N 107°37′11″ E	Warm temperate semi-humid	1000–1400	6–14	200–400
NW	42°55′15″ N 89°25′53″ E	Temperate continental	500–1000	9–10	<100
SW	31°74′24″ N 104°46′12″ E	Subtropical monsoon	250–1500	17–20	1200–1300

**Table 2:** Node attributes of soil microbial networks.

Node Species Name	Phylum	Degree	Degree Centrality	Closeness Centrality	Betweenness Centrality
unclassified_d*__Bacteria*	unclassified_Bacteria	16	0.32653	0.58333	0.03122
unclassified_o*_Rokubacteriales*	Methylomirabilota	14	0.28571	0.53846	0.02965
unclassified_o*_SBR1031*	Chloroflexi	13	0.26531	0.52688	0.01462
*Immundisolibacter*	Proteobacteria	20	0.40816	0.6125	0.05292
unclassified_f*_Comamonadaceae*	Proteobacteria	15	0.30612	0.53846	0.02702
*Acinetobacter*	Proteobacteria	18	0.36735	0.55682	0.01917
unclassified_o*_NRL2*	Proteobacteria	15	0.30612	0.53846	0.01706
*Blastococcus*	Actinobacteriota	18	0.36735	0.57647	0.02177
*Skermanella*	Proteobacteria	17	0.34694	0.56977	0.02152
*Nocardioides*	Actinobacteriota	9	0.18367	0.5	0.01287
*Arthrobacter*	Actinobacteriota	7	0.14286	0.44954	0.00216
unclassified_f*_Planococcaceae*	Firmicutes	14	0.28571	0.53846	0.04227
*Kocuria*	Actinobacteriota	20	0.40816	0.58333	0.05557
*Mycobacterium*	Actinobacteriota	8	0.16327	0.46226	0.0472
*Ellin6055*	Proteobacteria	17	0.34694	0.56977	0.0259
unclassified_f*_JG30-KF-CM45*	Chloroflexi	22	0.44898	0.62821	0.02783
*Truepera*	Deinococcota	21	0.42857	0.62025	0.02391
*Halomonas*	Proteobacteria	16	0.32653	0.55056	0.01995
unclassified_f*_AKYG1722*	Chloroflexi	17	0.34694	0.57647	0.01181
unclassified_f*_Longimicrobiaceae*	Gemmatimonadota	23	0.46939	0.64474	0.05901
unclassified_c*_bacteriap25*	Myxococcota	15	0.30612	0.55056	0.02146
*Sphingomonas*	Proteobacteria	16	0.32653	0.55056	0.0291
unclassified_o*_Saccharimonadales*	Patescibacteria	18	0.36735	0.55682	0.04731
unclassified_c*_Acidimicrobiia*	Actinobacteriota	16	0.32653	0.55682	0.04405
unclassified_c*_Gitt-GS-136*	Chloroflexi	9	0.18367	0.47115	0.0068
unclassified_f*_Vicinamibacteraceae*	Acidobacteriota	8	0.16327	0.47115	0.00629
unclassified_o*_Vicinamibacterales*	Acidobacteriota	7	0.14286	0.45794	0.00295
*Antarcticibacterium*	Bacteroidota	16	0.32653	0.56977	0.06202
*Iamia*	Actinobacteriota	8	0.16327	0.49	0.02301

## Data Availability

Restrictions apply to the availability of these data. Data were obtained from [majirbio] and are available [https://cloud.majorbio.com/page/tools/], with the permission of [majirbio].

## Supplementary Materials

The following supporting information can be downloaded at: https://www.mdpi.com/article/10.3390/microorganisms12112379/s1, Table S1: Physical and chemical properties of soil samples. Table S2: Total petroleum hydrocarbon content in soil samples. Table S3: Alpha diversity index of contaminated soil samples from different oil fields. Table S4: Relative abundance of primary metabolic pathways of samples.
