# Supplementary material for: Analysis of Microbial Community Heterogeneity and Carbon Fixation Capabilities in Oil-Contaminated Soils in Chinese Onshore Oilfields
Source: Microorganisms. 2024 Nov 20;12(11):2379. doi: 10.3390/microorganisms12112379 (PMC11596683; doi:10.3390/microorganisms12112379)
Supplement: Supplementary file 1 [file microorganisms-12-02379-s001.zip › microorganisms-3274802-supplementary.pdf]

Analysis of Microbial Community Heterogeneity and Carbon Fixation Capabilities in Oil-Contaminated Soils of Chinese Onshore Oilfields

Jiaya Song [1,2,†], Yakui Chen [3†✉], Yilei Han [3✉], Yunzhao Li [4], Zheng Liu [3], Xingchun Li [1,2], Diannan Lu [3✉], Chunmao Chen [4]

[1] State Key Laboratory of Petroleum Pollution Control, Beijing 102206, China

[2] CNPC Research Institute of Safety and Environmental Technology, Beijing 102206, China

[3] Key Lab of Industrial biocatalysis, Ministry of Education, Department of Chemical Engineering, Tsinghua University, Beijing 100084, China

[4] College of Chemical Engineering and Environment, China University of Petroleum-Beijing, Beijing 102206, China

[†] These authors contributed equally.

✉Corresponding Author

Email:chenyk1006@126.com(Y.C.);hanyl15@163.com(Y.H.);ludiannan@tsinghua.edu.cn (D.L)

Table S1. Physical and chemical properties of soil samples

Soil samples	pH	WC (%)	EC (ms/m)	NH_4^+-N (mg/kg)	NO_3^--N (mg/kg)	TN (g/kg)	TP (g/kg)	SOC (g/kg)
NW1	9.08±0.06	7.93±0.25	1273.33±56.86	3.74±0.36	7.12±0.61	0.51±0.15	0.75±0.03	3.37±0.21
NW2	8.56±0.16	4.17±0.15	469.33±33.95	34.80±1.70	56.20±2.65	0.77±0.21	0.88±0.06	5.37±0.32
NW3	9.15±0.05	9.17±0.15	360.67±32.87	< 0.25	14.20±1.75	0.40±0.23	0.63±0.07	3.03±0.35
NW4	8.47±0.09	3.83±0.06	128.00±3.00	9.90±0.44	25.37±0.64	0.62±0.25	0.72±0.03	5.00±0.10
SW1	8.42±0.00	11.30±0.00	24.7±0.00	1.01±0.00	2.84+0.00	0.42±0.00	0.58±0.00	53.50±1.06
SW2	8.74	12.60±0.00	18.1±0.00	< 0.25	2.72±0.00	0.41±0.00	0.58±0.00	14.13±0.80
SW3	8.74	12.20±0.00	9.6±0.00	< 0.25	8.51±0.00	0.25±0.00	0.62±0.00	38.23±2.54
SW4	8.41	13.30±0.00	19.5±0.00	< 0.25	6.37±0.00	0.40±0.00	0.62±0.00	13.93±0.67

SW5	8.04±0.00	12.90±0.00	21±0.00	< 0.25	2.16±0.00	0.78±0.00	0.6±0.00	14.93±1.02
C1	8.62±0.03	3.53±0.21	91.37±0.31	9.08±0.70	30.50±0.46	0.78±16.07	0.55±0.01	49.10±11.69
C2	8.94±0.03	5.03±0.21	28.53±0.40	8.34±0.54	13.33±0.31	1.73±20.00	0.64±0.01	39.43±7.96
C3	8.42±0.06	7.13±0.15	26.73±0.47	9.52±0.84	5.31±0.18	1.61±86.22	0.64±0.01	31.97±6.29
C4	8.99±0.06	5.73±0.15	226.67±3.21	10.93±1.04	173.00±2.65	0.74±41.88	0.49±0.01	34.23±3.60
C5	9.22±0.05	5.77±0.15	233.67±0.58	9.35±0.70	205.67±1.15	0.71±52.56	0.27±0.01	-
C6	8.45±0.04	0.33±0.06	42.53±0.25	13.93±1.43	7.07±0.35	0.85±37.16	0.19±0.01	-
C7	8.25±0.04	0.37±0.06	53.23±0.49	8.67±0.69	1.31±0.21	0.77±38.42	0.37±0.01	-
E1	8.19±0.16	27.58±0.09	40.63±0.50	3.12±0.07	3.89±0.05	2.05±0.07	0.42±0.13	80.55±0.78
E2	9.06±0.06	25.90±0.30	41.80±0.50	1.76±0.05	0.70±0.06	1.30±0.00	1.82±1.53	60.40±0.14
E3	8.83±0.08	23.27±0.97	25.87±0.51	1.67±0.11	0.85±0.06	1.85±0.07	2.80±2.19	92.95±2.76

E4	8.31±0.23	26.52±0.47	62.73±0.67	2.29±0.01	0.82±0.06	1.50±0.00	0.63±0.26	67.60±1.27
E5	8.49±0.37	26.77±0.38	4.47±0.06	1.71±0.03	0.78±0.07	1.25±0.07	3.67±0.54	61.80±0.42
E6	8.37±0.37	27.41±0.34	43.67±0.25	1.58±0.01	0.66±0.06	1.45±0.07	3.37±0.03	70.30±0.85
E7	8.65±0.10	24.45±0.44	35.43±0.51	2.62±0.05	0.68±0.06	2.15±0.35	2.62±3.42	97.40±11.03
E8	8.81±0.30	23.59±0.75	32.07±0.50	1.27±0.04	0.54±0.04	1.40±0.00	3.05±1.80	65.35±0.49
E9	8.82±0.04	23.59±0.36	31.50±0.46	1.49±0.05	0.55±0.05	1.15±0.07	3.46±0.85	52.30±2.55
E10	8.79±0.08	16.61±0.82	39.07±0.51	1.50±0.02	0.67±0.03	1.30±0.00	2.81±1.94	47.50±0.00
E11	8.85±0.06	9.00±0.00	39.17±0.35	1.15±0.01	0.63±0.04	3.15±0.07	0.16±0.23	120.75±5.02

Table S2. Total petroleum hydrocarbon content in soil samples

Site	Description of sample	Total petroleum hydrocarbon content
E	E1-E11	417.20±66.19;444.10±17.11;478.50±41.72; 784.85±514.28;616.30±297.41;43.04±9.72; 44.18±3.11;247.53±283.51;86.43±29.06; 432.60±21.78;1027.75±15.91
C	C1-C7	1636.67±179.54;3950.00±230.65;2726.67±447.92; 896.33±172.37;509.00±74.36;9453.33±1560.30; 20433.33±1001.67
NW	NW1-NW4	117.00±8.89; 323.00±70.66; 224.00±14.73; 270.00±27.73
SW	SW1-SW5	1420.00±0.00; 6830±0.00; 1600.00±0.00; 426.00±0.00; 770.00±0.00

Table S3. Alpha diversity index of contaminated soil samples from different oil fields

Study region	Description of sample	Alpha diversity index		
		Simpson	Ace	Coverage
Eastern oilfield	E1	0.216	54.583	0.999
	E2	0.143	57.484	0.999
	E3	0.433	68.144	0.999
	E4	0.292	44.654	0.999
	E5	0.330	43.876	0.999
	E6	0.254	35.207	0.999
	E7	0.452	56.020	0.999
	E8	0.4290	59.133	0.999
	E9	0.199	54.820	0.999
	E10	0.180	50.413	0.999
	E11	0.167	33.000	1
Central Oilfield	C1	0.195	34.371	0.999
	C2	0.225	28.398	0.999
	C3	0.249	28.455	0.999
	C4	0.252	30.998	0.999
	C5	0.226	30.654	0.999
	C6	0.304	26.418	0.999
	C7	0.376	22.395	0.999
Northwestern Oilfield	NW1	0.202	22.594	0.999
	NW2	0.172	29.387	0.999
	NW3	0.217	30.686	0.999
	NW4	0.169	24.642	0.999
Southwestern oilfield	SW1	0.396	45.433	0.999
	SW2	0.259	30.000	1
	SW3	0.278	24.619	0.999
	SW4	0.244	28.504	0.999
	SW5	0.254	29.380	0.999

Table S4. Relative abundance of primary metabolic pathways of samples.

Description of Sample	Prediction Pathway Level 1 Relative abundance（%）					
	Cellular Processes	Environmental Information Processing	Genetic Information Processing	Human Diseases	Metabolism	Organismal Systems
E1	4.50%	5.50%	6.66%	3.62%	77.73%	2.00%
E2	4.13%	5.78%	6.82%	3.77%	77.52%	1.99%
E3	4.02%	5.56%	6.77%	3.68%	77.96%	1.99%
E4	4.56%	5.52%	6.58%	3.35%	78.12%	1.87%
E5	4.29%	5.30%	6.98%	3.38%	78.16%	1.88%
E6	4.59%	5.46%	6.95%	3.59%	77.50%	1.91%
E7	4.60%	5.45%	7.09%	3.53%	77.50%	1.84%
E8	4.03%	5.19%	6.26%	3.38%	79.11%	2.03%
E9	4.75%	5.78%	6.79%	3.98%	76.71%	1.99%
E10	4.25%	5.22%	6.45%	3.37%	78.80%	1.91%
E11	4.35%	5.10%	6.93%	3.27%	78.53%	1.83%
C1	4.42%	4.83%	7.10%	3.26%	78.64%	1.75%
C2	4.63%	5.12%	6.62%	3.61%	78.17%	1.86%
C3	4.77%	5.22%	6.55%	3.71%	77.87%	1.88%
C4	4.86%	5.51%	6.56%	3.73%	77.50%	1.84%
C5	4.75%	5.43%	6.58%	3.69%	77.70%	1.84%
C6	3.95%	5.03%	6.08%	3.15%	79.90%	1.90%
C7	4.11%	5.19%	5.71%	3.43%	79.65%	1.90%
NW1	3.94%	4.89%	6.78%	3.18%	79.39%	1.82%
NW2	4.59%	6.20%	6.46%	3.08%	77.85%	1.81%
NW3	4.40%	5.22%	6.86%	3.34%	78.33%	1.84%
NW4	4.25%	5.34%	6.39%	3.57%	78.52%	1.93%
SW1	4.80%	6.02%	5.89%	3.86%	77.34%	2.09%
SW2	3.58%	4.63%	6.47%	2.90%	80.54%	1.86%
SW3	3.87%	4.77%	6.41%	3.35%	79.67%	1.93%
SW4	4.55%	5.04%	6.29%	3.81%	78.37%	1.94%
SW5	3.85%	4.80%	6.37%	3.31%	79.73%	1.94%
